# A framework of biomarkers for skin aging: a consensus statement by the Aging Biomarker Consortium

**DOI:** 10.1093/lifemedi/lnag032

**Published:** 2026-10-05

**Authors:** Aging Biomarker Consortium, Yiya Zhang, Mengting Chen, Zhijin Li, Yingxue Huang, Qian Zhao, Ting Chen, Xiang Chen, Yong Cui, Zhili Deng, Yanan Du, Xinhua Gao, Jing-Dong Jackie Han, Yujun Hou, Zhe Jian, Di Li, Hang Li, Zhimiao Lin, Hong Liu, Wenjing Luo, Yuling Shi, Liangdan Sun, Juan Tao, Yang Wang, Fang Wang, Haijing Wu, Ran Xiao, Yong Yang, Nanze Yu, Bing Zhang, Furen Zhang, Lingjuan Zhang, Weiqi Zhang, Ming Zhao, Guang-Hui Liu, Gang Pei, Xiao Long, Jing Qu, Ji Li

**Affiliations:** Department of Dermatology, Hunan Key Laboratory of Aging Biology, Xiangya Hospital, Central South University, National Clinical Research Center for Geriatric Diseases (XiangYa Hospital of Central South University), Changsha 410008, China; FuRong Laboratory, Changsha 410008, China; Department of Dermatology, Hunan Key Laboratory of Aging Biology, Xiangya Hospital, Central South University, National Clinical Research Center for Geriatric Diseases (XiangYa Hospital of Central South University), Changsha 410008, China; FuRong Laboratory, Changsha 410008, China; Department of Plastic and Aesthetic Surgery, Center for Regenerative Medicine & Plastic Surgery Research, Beijing Key Laboratory of Aging Regulation and Translational Intervention for Skin and Soft Tissue, Peking Union Medical College Hospital, Chinese Academy of Medical Sciences & Peking Union Medical College, Beijing 100032, China; Department of Dermatology, Hunan Key Laboratory of Aging Biology, Xiangya Hospital, Central South University, National Clinical Research Center for Geriatric Diseases (XiangYa Hospital of Central South University), Changsha 410008, China; FuRong Laboratory, Changsha 410008, China; Advanced Innovation Center for Human Brain Protection, National Clinical Research Center for Geriatric Disorders, Aging Translational Medicine Center, Beijing Municipal Geriatric Medical Research Center, Beijing Key Laboratory of Environment and Aging, Xuanwu Hospital Capital Medical University, Beijing 100053, China; National Institute of Biological Sciences, Beijing 102206, China; Tsinghua Institute of Multidisciplinary Biomedical Research, Tsinghua University, Beijing 102206, China; FuRong Laboratory, Changsha 410008, China; Department of Dermatology, Xiangya Hospital, Central South University, National Clinical Research Center for Geriatric Diseases (XiangYa Hospital of Central South University), Changsha 410008, China; Department of Dermatology, China-Japan Friendship Hospital, Beijing 100029, China; Department of Dermatology, Hunan Key Laboratory of Aging Biology, Xiangya Hospital, Central South University, National Clinical Research Center for Geriatric Diseases (XiangYa Hospital of Central South University), Changsha 410008, China; FuRong Laboratory, Changsha 410008, China; School of Biomedical Engineering, Tsinghua Medicine, Tsinghua University, Beijing 100084, China; Department of Dermatology, The First Hospital of China Medical University, Shenyang 110001, China; Peking-Tsinghua Center for Life Sciences, Academy for Advanced Interdisciplinary Studies, Center of Quantitative Biology (CQB), Peking University, Beijing 100871, China; Institute for Regenerative Medicine, State Key Laboratory of Cardiology and Medical Innovation Center, Shanghai East Hospital, Frontier Science Center for Stem Cell Research, School of Life Sciences and Technology, Tongji University, Shanghai 200092, China; Department of dermatology, Xijing Hospital, The Fourth Military Medical University, Xi’an 710032, China; School of Chemistry and Molecular Engineering, East China Normal University, Shanghai 200241, China; Department of Dermatology, Peking University First Hospital, Beijing 100034, China; Dermatology Hospital, Southern Medical University, Guangzhou 510091, China; Dermatology Hospital of Shandong First Medical University, Jinan 250022, China; Department of Occupational and Environmental Health, School of Public Health, Ministry of Education Key Laboratory of Hazard Assessment and Control in Special Operational Environments, Fourth Military Medical University, Xi’an 710032, China; Department of Dermatology, Shanghai Skin Disease Hospital, Tongji University School of Medicine, Shanghai 200443, China; Health Science Center, North China University of Science and Technology, Tangshan 063210, China; Department of Dermatology, Union Hospital, Tongji Medical College, Huazhong University of Science and Technology, Wuhan 430030, China; Department of Dermatology and Venereology, Peking University First Hospital, Beijing 100034, China; Dermatology Hospital, Southern Medical University, Guangzhou 510091, China; Department of Dermatology, Second Xiangya Hospital, Central South University, Changsha 410011, China; Research Center of Plastic Surgery Hospital, Chinese Academy of Medical Sciences and Peking Union Medical College, Beijing 100144, China; Hospital for Skin Diseases, Institute of Dermatology, Chinese Academy of Medical Sciences and Peking Union Medical College, Nanjing 210042, China; Department of Plastic and Aesthetic Surgery, Center for Regenerative Medicine & Plastic Surgery Research, Beijing Key Laboratory of Aging Regulation and Translational Intervention for Skin and Soft Tissue, Peking Union Medical College Hospital, Chinese Academy of Medical Sciences & Peking Union Medical College, Beijing 100032, China; School of Life Sciences, Westlake University, Hangzhou 310024, China; Dermatology Hospital of Shandong First Medical University, Jinan 250022, China; State Key Laboratory of Cellular Stress Biology, School of Pharmaceutical Sciences, Xiamen University, Xiamen 361102, China; Beijing Key Laboratory of Intelligent Governance and Application of Biological Big Data, China National Center for Bioinformation and Beijing Institute of Genomics, Chinese Academy of Sciences, Beijing 100101, China; Hospital for Skin Diseases, Institute of Dermatology, Chinese Academy of Medical Sciences and Peking Union Medical College, Nanjing 210042, China; State Key Laboratory of Organ Regeneration and Reconstruction, Human Organ Physiopathology Emulation System, Institute of Zoology, Chinese Academy of Sciences, Beijing 100101, China; Collaborative Innovation Center for Brain Science, School of Life Science and Technology, Tongji University, Shanghai 200092, China; Department of Plastic and Aesthetic Surgery, Center for Regenerative Medicine & Plastic Surgery Research, Beijing Key Laboratory of Aging Regulation and Translational Intervention for Skin and Soft Tissue, Peking Union Medical College Hospital, Chinese Academy of Medical Sciences & Peking Union Medical College, Beijing 100032, China; State Key Laboratory of Organ Regeneration and Reconstruction, Human Organ Physiopathology Emulation System, Institute of Zoology, Chinese Academy of Sciences, Beijing 100101, China; University of Chinese Academy of Sciences, Beijing 100049, China; Beijing Institute for Stem Cell and Regenerative Medicine, Beijing 100101, China; Department of Dermatology, Hunan Key Laboratory of Aging Biology, Xiangya Hospital, Central South University, National Clinical Research Center for Geriatric Diseases (XiangYa Hospital of Central South University), Changsha 410008, China; FuRong Laboratory, Changsha 410008, China

## Abstract

Skin is the largest organ of the human body and the first line of defense against the external environment. With the rapid growth of the older population in China, significant challenges have emerged in the prevention and management of age-associated diseases, while skin aging is closely associated with the aging of multiple organs. To address these challenges, the Aging Biomarker Consortium (ABC) has established an expert consensus on biomarkers of skin aging through a review of the literature and consultation with scientists and clinicians. This consensus provides a comprehensive evaluation of the key changes, characteristics, and biomarkers related to skin aging and proposes a systematic framework that categorizes these biomarkers into structural, functional, and humoral biomarkers. Within each dimension, the ABC recommends biomarkers supported by varying levels of clinical and experimental evidence for assessing both physiological and pathological changes in skin tissue during aging, aiming to establish a foundation for the prediction, diagnosis, early detection, and treatment of skin aging and related diseases, with the ultimate goal of improving skin tissue health and promoting healthy aging in elderly populations both in China and worldwide.

## Introduction

As the largest organ of the human body and the first line of defense against the external environment, the skin plays an irreplaceable role in maintaining physical barrier function, preventing pathogen invasion, and resisting mechanical injury [1, 2]. Skin aging includes intrinsic (natural) aging and extrinsic (environmental) aging, and refers to the process in which the structure and function of skin tissue progressively decline under the influence of genetic factors, cumulative time, and environmental exposures (such as ultraviolet radiation and pollutants) [3]. Structural changes are manifested as abnormalities in epidermal and dermal thickness, flattening of the rete ridges, reduction of subcutaneous adipose tissue, and degenerative changes in skin appendages (hair follicles, sweat glands, sebaceous glands, and nails) [4–6]. Functional changes include impaired barrier function, delayed wound healing, weakened immune responses, and reduced sensory function.

Skin aging is not only the most visible external manifestation of organismal aging, affecting psychosocial health and quality of life, but is also closely associated with systemic aging processes. Skin aging phenotypes, including facial appearance and skin wrinkling, have been associated with familial longevity, cardiovascular disease risk, and mortality [7, 8]. In addition, the skin’s neuroendocrine-immune system may contribute to both local and systemic homeostasis [9]. The accumulation of p16^+^ cells in the skin is positively correlated with biomarkers of immune aging and biological age [10, 11]. Therefore, assessing the degree of skin aging is not only critical for monitoring the condition of the skin itself, but also provides an important basis for predicting an individual’s biological age and the risk of systemic aging-related diseases (such as cardiovascular diseases and neurodegenerative disorders), which is of substantial clinical significance for achieving healthy aging.

According to the latest official demographic data, the population aged 60 years and above in China reached 310.31 million (22.0% of the total population) by the end of 2024; population aging, together with the growing burden of chronic diseases and multimorbidity, poses major challenges to China’s public health and healthcare systems. In this context, exploring reliable biomarkers that can objectively assess the degree of skin aging will not only help establish an individualized skin aging assessment system, but may also provide new strategies for the early intervention of age-related diseases, thereby promoting the application of precision medicine in the field of anti-aging.

On December 13, 2025, the Aging Biomarker Consortium (ABC) [12–23] convened experts in skin research and aging research at a workshop held in Changsha (2025). Based on published literature, peer-reviewed research foundations, evidence-based medical data, and expert opinions, the consortium established an expert consensus recommending biomarkers that reflect skin aging. These biomarkers are intended to assess individual skin tissue aging status and rate, predict risks of skin aging-related diseases, and provide a scientific evaluation system for delaying and intervening in skin aging and its associated diseases, ultimately aiming to extend lifespan and health span.

## Recommended methodology for biomarkers for skin aging

A systematic literature search using major bibliographic databases, including PubMed (including MEDLINE) and the Cochrane Library, as well as other sources relevant to the scope of this consensus, was performed to identify studies published before November 2025. Based on available evidence, online collaborative discussions, and the collective research expertise of ABC members, a series of key questions concerning skin aging biomarkers were identified. Candidate biomarkers of skin aging were then critically evaluated and extensively discussed by the expert panel, culminating in an expert consensus at a validation meeting on skin aging biomarkers. All recommendations were subjected to comprehensive review and repeated discussion among ABC members to ensure that the final document reflected broad expert perspectives and multidisciplinary considerations.

The grading of the level of evidence and strength of recommendations in this consensus was based on internationally recognized standards, as shown in Table 1.

**Table 1.** Classification and definition of levels of evidence and levels of recommendation

Class (strength) of recommendation	Level (quality) of evidence
**Class I (strong) benefit >>> risk**	**Level A**
**Suggested phrases for writing recommendation** **Recommended/Indicated** **Evidence and/or general agreement that a given treatment or procedure is beneficial, useful and effective**	Data derived from multiple randomized clinical trials or meta-analyses
**Class IIa (moderate) benefit >> risk**	**Level B**
**Suggested phrases for writing recommendation** **Should be considered** **Weight of evidence/opinion is in favor of usefulness/efficacy**	Data derived from a single randomized clinical trial or large non-randomized studies
**Class IIb (weak) benefit ≥ risk**	**Level C**
**Suggested phrases for writing recommendation** **May be considered** **Usefulness/efficacy is less well established by evidence/opinion**	Consensus of expert opinion, and/or small studies, retrospective studies, registries
**Class III (strong) risk > benefit** **Suggested phrases for writing recommendation** **Not recommended** **Evidence/general agreement that the given treatment/procedure is not useful/effective and may, in some cases, be harmful.**	**Note:** COR and LOE are determined independently (any COR may be paired with any LOE). COR, Class of recommendation; LOE, Level of evidence.

## Classification and clinical application of skin aging biomarkers

Skin aging involves multidimensional alterations at the molecular, cellular, organ, individual, and population levels. Skin aging biomarkers refer to indicators that accurately reflect “true skin age,” “skin structure,” and “skin function.” These biomarkers can be used to assess the degree and rate of skin aging, evaluate disease risk, and monitor the efficacy of anti-aging interventions. This consensus identifies biomarkers of skin aging across three dimensions: structural, functional, and humoral biomarkers (Fig. 1) for reference in clinical practice and future research.

**Figure 1.**
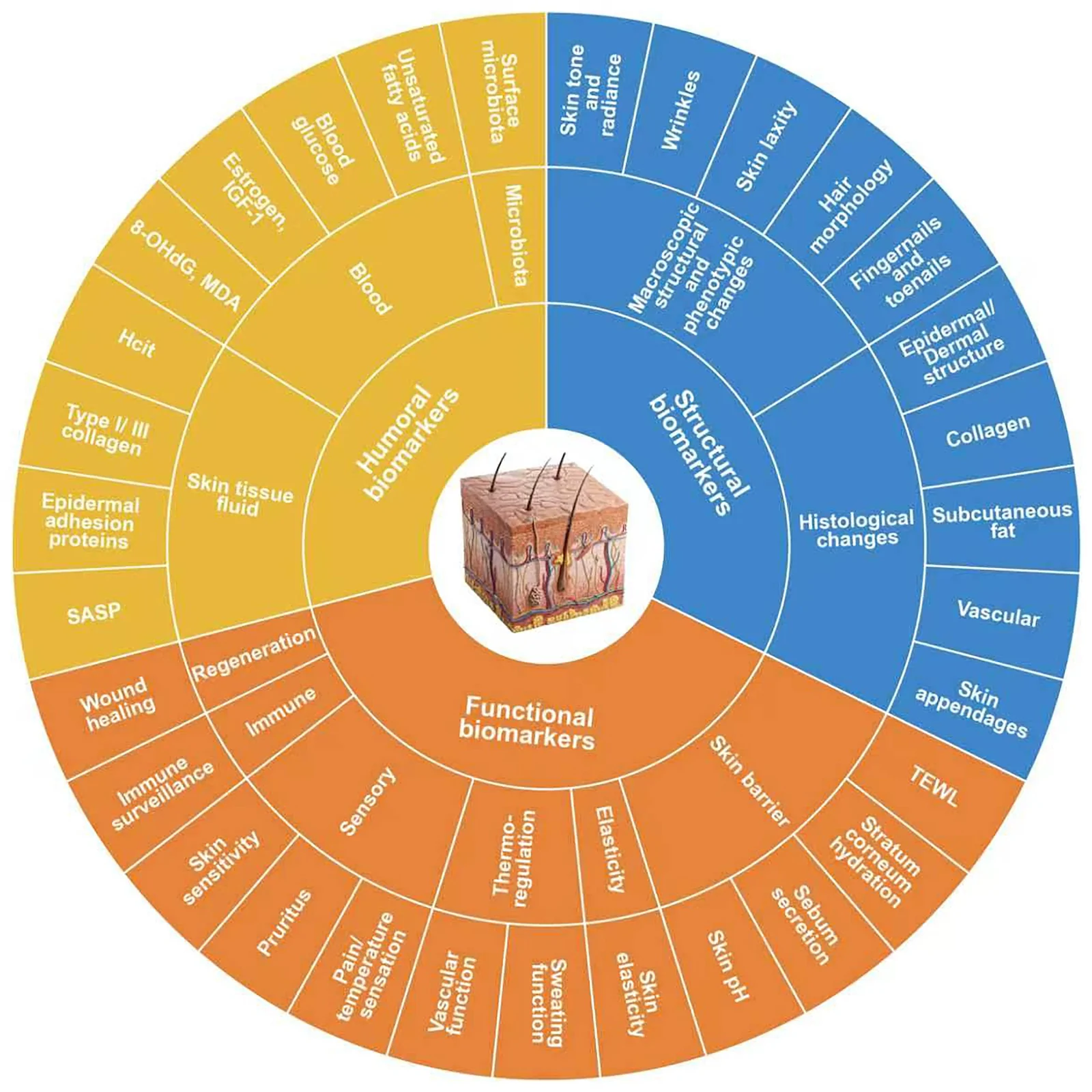
Biomarker framework for skin tissue aging. The proposed framework for skin aging consists of three dimensions: structural, functional, and humoral biomarkers. The recommended biomarkers cover multidimensional and multilevel changes associated with skin aging. These biomarkers show promising potential for widespread application in routine clinical practice. However, it should be emphasized that additional validation studies are required to evaluate the efficacy of these biomarkers in assessing skin aging.

## Structural biomarkers

Skin aging is accompanied by substantial alterations in tissue architecture, and these structural changes often provide the pathological basis for the development of cutaneous disorders. Therefore, a comprehensive understanding of the morphological characteristics, microstructural alterations, and related molecular biomarkers of skin aging is essential for accurately evaluating skin aging and predicting disease risk.

### Macroscopic structural and phenotypic changes in aging skin

#### Skin tone, radiance, and texture

Aging skin commonly presents with dullness, reduced overall brightness, dyspigmentation, rough texture, and diminished radiance. Representative pigmentary changes include hyperpigmented lesions and hypopigmented lesions, such as seborrheic keratoses and idiopathic guttate hypomelanosis. These visible alterations primarily reflect disruption of pigment homeostasis, including melanin synthesis, transfer, and degradation, together with structural abnormalities in keratinocytes, such as irregular cellular morphology and organization and weakened intercellular cohesion.

In chronically photo-exposed areas, repeated cumulative ultraviolet exposure may maintain melanocytes in a relatively persistent activated state compared with non-exposed aged skin, thereby increasing susceptibility to focal hyperpigmentation [24]. By contrast, in non-exposed areas, aging-associated melanocyte dysfunction and reduced melanin content in the basal layer may result in a paler complexion and reduced radiance. In parallel, reduced dermal microvascular perfusion contributes to decreased skin redness and a pale appearance, whereas irregular keratinocyte arrangement and weakened stratum corneum integrity contribute to surface roughness. Collectively, these changes constitute characteristic phenotypic features of aging skin [25, 26].

These changes can be quantified using objective instruments. Spectrophotometric colorimetry (e.g., Chromameter) can be used to assess skin lightness and color difference, whereas Mexameter can quantify erythema index (EI) and melanin index (MI). Multispectral imaging systems such as VISIA may further quantify skin roughness and other surface features. Because the relationship between melanin index and age is strongly influenced by cumulative sun exposure, these parameters should be interpreted in conjunction with photo-exposure history and other morphological markers. Measures of skin roughness should also be interpreted alongside indices of skin barrier function.

#### Wrinkles

Wrinkles are among the most typical textural manifestations of structural skin aging and primarily reflect progressive disruption of the dermal collagen and elastic fiber networks. Collagen and elastic fibers undergo fragmentation, rupture, and abnormal remodeling, leading directly to visible linear or furrow-like surface changes. In some cases, reduced structural support around follicular ostia may also contribute to the appearance of enlarged pores. Early structural wrinkles typically present as fine dynamic lines associated with facial movement and gradually progress to deeper static lines and coarse furrows visible at rest.

A population-based study from southeastern China, in which three physicians clinically assessed periorbital and forehead wrinkles, graded wrinkle severity according to wrinkle depth and the influence of facial expression. The study showed that the prevalence of facial wrinkles increased significantly with age, from 33% in the young group to 87.8% in the middle-aged group and 100% in the older group [27]. In contrast, wrinkles are less prominent in sun-protected areas. In clinical practice, wrinkle severity is commonly assessed using visual rating scales, including the Glogau photoaging classification, the Fitzpatrick Wrinkle Scale, the Merz Aesthetics Scale (MAS), and the Lemperle wrinkle assessment scale. To avoid confusion, it should be noted that the Fitzpatrick Wrinkle Scale specifically assesses wrinkle severity, whereas the Fitzpatrick Skin Type classification is a separate system used to evaluate an individual’s susceptibility to photodamage. Among these, MAS has been validated across multiple countries and has shown good reliability and validity for comprehensive facial aging assessment.

Objective instrumental techniques provide quantitative evaluation of wrinkles and skin texture. Three-dimensional skin surface profilometry, such as PRIMOS phase-shifting optical profilometry, can characterize wrinkle topography and generate quantitative parameters including wrinkle depth, length, and volume, thereby providing an objective index of structural degeneration in aging skin. Studies have shown that PRIMOS-measured wrinkle depth, including crow’s feet depth, is strongly associated with age; for every 10-year increase in age, wrinkle depth increases by approximately 50 μm, corresponding to an approximately one-grade increase in subjective wrinkle score [28]. PRIMOS is also sensitive to subtle improvements induced by anti-aging interventions, such as significant reductions in wrinkle depth and volume following oral citrus extract supplementation [29]. VISIA imaging systems can provide wrinkle counts, area scores, and a composite “skin age,” which is highly correlated with chronological age (*r *= 0.83) and demonstrates good reproducibility, with repeat-test variation of approximately 3% [30]. In addition, newer three-dimensional surface imaging approaches, including stereophotogrammetry and structured-light scanning, allow precise quantification of wrinkle depth, length, area, and volume for longitudinal evaluation and monitoring of skin aging [31, 32].

#### Skin laxity

Increased skin laxity reflects degenerative changes in the multilayer dermal-subcutaneous support system. Reductions in dermal collagen and elastic fibers, together with their abnormal remodeling, as well as laxity of the superficial fascia, retaining ligaments, and fibrous septa within subcutaneous fat, contribute to soft-tissue descent and contour sagging. Clinically, these changes may manifest as prominent lower eyelid bags, deepening of the nasolabial folds, and loss of mandibular contour definition.

Skin laxity can be quantified by combining standardized clinical grading scales with objective digital imaging parameters. For example, jawline sagging may be evaluated using photo-numeric grading scales. Alternatively, multi-angle photography and three-dimensional stereophotogrammetry, including structured-light or stereophotographic systems, can be used to measure soft-tissue descent distance at representative sites such as the cheeks and jawline, as well as changes in contour curvature, contour definition, and the area or volume of sagging regions. These approaches enable reproducible and comparable structural assessment of age-related skin laxity [33].

#### Hair

Hair graying is one of the most visible signs of aging. Studies have shown that, across different ethnic populations, approximately 6%–23% of individuals have at least 50% gray hair by the age of 50 years [34]. In addition to scalp hair, the eyebrows, beard, and body hair may also undergo graying with age. However, hair-related traits are strongly influenced by genetic background and ethnicity. Some individuals retain a substantial proportion of pigmented hair even in their sixth or seventh decade of life, whereas others develop marked hair graying in their thirties, consistent with premature canities.

In addition to pigmentary changes, hair density, shaft diameter, and total hair number also decline with age. These features are typically evaluated by visual inspection or dermoscopy, both of which are simple and inexpensive. However, because hair characteristics are strongly affected by heredity and endocrine factors, particularly androgens, they are of limited value as precise quantitative markers of aging and are better regarded as visible supportive signs.

Hair mechanical strength also declines with age. This parameter can be measured by automated tensile testing. Typically, multiple hairs, for example, 10 shafts, are collected from the vertex near the scalp, and individual hairs are mounted between the clamps of a tensile testing device and stretched at a constant rate until breakage. The maximum force required to fracture the hair fiber is recorded as an index of hair strength [35]. Overall, hair-related parameters are better considered adjunctive indicators in skin aging assessment and should be interpreted in combination with skin imaging, physiological function, histology, and molecular biomarkers to minimize bias introduced by genetic background, endocrine status, and external factors such as grooming, dyeing, and perming.

#### Nails

As skin appendages, nails also undergo substantial age-related changes. Major alterations include progressive thinning of the nail plate, increased brittleness, and a decline in nail growth rate. From the age of 25 years onward, nail growth decreases by approximately 0.5% per year [36]. Morphologically, the longitudinal curvature of the nail gradually decreases, whereas transverse convexity increases, leading to a flatter appearance. The nail surface becomes rougher and more prone to fissuring, lamellar splitting, and longitudinal ridging; the lunula becomes smaller or may disappear, and nail gloss diminishes. In parallel, degenerative changes such as vascular wall thickening may occur in the dermal vasculature of the nail bed, suggesting age-related impairment of nail-bed microcirculation and vascular elasticity [37].

Epidemiological studies indicate that approximately 34% of older adults have increased fingernail or toenail brittleness, with a reported prevalence of approximately 40% in men and 26% in women. Some older individuals may also develop nail plate thickening and deformation, particularly in the toenails, as a consequence of poor peripheral circulation or chronic trauma [38]. In addition, periungual skin may show age-related alterations in color and texture similar to those observed elsewhere in the skin and may therefore provide additional visible clues to organismal aging.

During assessment, potential confounders should be carefully excluded, as fungal infection, nail dystrophy, and other nail disorders may produce similar changes. Dermoscopy is an effective noninvasive tool for evaluating morphological, pigmentary, and vascular changes in the nail plate, nail bed, and periungual tissues.

### Histological changes in the skin

#### Epidermis

As the outermost defensive barrier of the skin, the epidermis undergoes substantial structural and functional decline during aging. It is composed predominantly of keratinocytes arranged, from superficial to deep, into the stratum corneum, stratum granulosum, stratum spinosum, and stratum basale, and also contains melanocytes, Langerhans cells, and other specialized cell populations. With increasing age, overall epidermal thickness decreases significantly, accompanied by reduced cellular density in the basal and spinous layers. Studies have shown that, across individuals aged 20–80 years, epidermal thickness declines progressively with age, with an average decrease of 7.2% per decade in men and 5.7% in women [39]. By contrast, epidermal thickness may increase in photoaged skin, indicating that intrinsic aging and photoaging differ in their effects on epidermal morphology. These structural alterations can be evaluated noninvasively using imaging modalities such as reflectance confocal microscopy (RCM), optical coherence tomography (OCT), and high-frequency ultrasound [40].

Keratinocytes are the principal cells responsible for maintaining epidermal barrier integrity, and age-related changes in these cells are important indicators of epidermal functional decline. With age, keratinocyte mitotic activity decreases markedly, resulting in a slower rate of epidermal turnover [41]. In parallel, keratinocytes enlarge, become morphologically irregular, and exhibit weakened intercellular cohesion, thereby compromising stratum corneum integrity, and reducing resistance to external insults. Clinically, these changes manifest as dryness, roughness, and scaling [42]. Such alterations can be directly visualized by RCM, including disorganized keratinocyte architecture and increased variability in cell size in aged skin.

Melanocyte number and distribution also change with age, and the capacity for melanin synthesis becomes regionally dysregulated. In photo-exposed skin, abnormal melanocyte activity may increase local melanin production, resulting in hyperpigmented lesions such as solar lentigines; in non-exposed skin, melanocyte number declines substantially, contributing to an overall lightening of skin color [24, 43]. RCM can visualize melanocyte morphology and distribution and reveal aging-related changes in pigment network architecture, thereby providing imaging support for pigment-associated aging phenotypes.

Langerhans cells are key antigen-presenting cells in the epidermis. Their density peaks at approximately 20–30 years of age and then gradually declines with advancing age [44]. Aging is associated not only with reduced cell number but also with morphological alteration. In intrinsic aging, nuclei enlarge while dendritic structures remain relatively intact; in photoaging, dendrites are reduced, Birbeck granules are lost, and antigen-presenting function declines substantially [45]. These degenerative changes may impair cutaneous immune surveillance and represent important features of epidermal immunosenescence. However, assessment currently depends largely on invasive histologic staining or electron microscopy, limiting routine clinical application.

#### Dermal-epidermal junction

The dermal-epidermal junction (DEJ) forms the interface between the epidermis and dermis and is composed of complex extracellular matrix (ECM) elements, including collagen, elastic fibers, and laminin. Together, these components maintain stable adhesion between the epidermis and dermis and support epidermal cell attachment, migration, and differentiation [46]. In young skin, the DEJ displays a characteristic undulating or rete-like architecture. During aging, however, the DEJ undergoes marked structural and functional remodeling.

First, the undulating architecture progressively flattens, reducing the contact surface area between the epidermis and dermis and thereby impairing nutrient exchange and waste clearance across the interface. Second, collagen, laminin, and other basement membrane zone components decrease, causing thinning and discontinuity of the basement membrane, while the underlying dermis becomes stiffer; together, these changes weaken resistance to shear stress [47]. Third, hemidesmosomes decrease in number and become structurally abnormal, further reducing adhesion strength between the epidermis and dermis. These age-related changes may vary by anatomical site. For example, dermal-epidermal attachment is inherently weaker at mucocutaneous junctions, which may make these regions more susceptible to atrophic fine lines with age. Histologic measurement remains the gold standard for evaluating DEJ flattening. However, RCM and related imaging techniques provide noninvasive *in vivo* visualization of DEJ undulation and allow quantitative measurement of interpapillary distance and papillary height [48].

#### Dermis

The dermis provides the major structural support and elastic resilience of the skin, and age-related loss of dermal volume and organization is a fundamental feature of skin aging. The dermis is composed primarily of ECM constituents, including collagen, elastin, and glycosaminoglycans, and contains fibroblasts, blood vessels, lymphatic vessels, and other stromal elements. With age, dermal thickness decreases, ECM homeostasis becomes disrupted, and fibroblast function declines, together giving rise to hallmark aging phenotypes such as laxity, reduced elasticity, and wrinkle formation [39, 49, 50].

Dermal thinning is one of the most prominent structural changes in aging skin. Studies have shown that dermal thickness declines by approximately 6% per decade in both men and women [39], providing a major histological basis for impaired dermal structural support and increased laxity. This thinning is driven largely by ECM loss and remodeling. The major ECM components, collagen and elastic fibers, undergo marked age-related quantitative and structural alterations. Fragmentation and reduction of collagen fibers, together with excessive deposition of abnormal elastic material, are characteristic features of aged skin [51]. Total dermal collagen content decreases by approximately 1% annually with age, with particularly pronounced reductions in type I and type III collagen, thereby weakening mechanical support and elasticity [52]. In parallel, collagen bundles become increasingly disorganized, fragmented, and ruptured, further compromising tissue resilience [50]. These alterations affect not only skin appearance but also susceptibility to mechanical injury.

Such changes can be quantified using advanced imaging approaches, including multiphoton microscopy and second harmonic generation (SHG) imaging. For example, three-dimensional multiphoton systems that combine two-photon excited fluorescence lifetime imaging microscopy (2PEF-FLIM) with SHG enable quantitative assessment of collagen and elastin architecture. Using these methods, aged skin has been shown to exhibit increased elastic fiber density in the superficial dermis, reduced collagen–derived SHG signal, and a lower SHG/autofluorescence ratio, also known as the SHG-to-autofluorescence aging index of dermis SAAID. In photo-exposed regions such as the forearm and face, the elastic fiber network increases with age and changes from a slender, orderly arrangement in youth to a dense, diffuse architecture in old age. At the same time, the degree of collagen–elastin interweaving, measured by the normalized imbrication index (ImbrN), increases with age, reflecting abnormal remodeling of the collagen–elastic network [53]. Importantly, however, some studies suggest that papillary dermal collagen density remains relatively stable during intrinsic aging in Asian populations, whereas it declines substantially with age in White populations [54]. This observation indicates that caution is warranted when applying collagen SHG as a skin aging biomarker in Asian cohorts.

High-frequency ultrasound also provides noninvasive assessment of dermal ECM changes. In young skin, the dermis appears as a relatively uniform hyperechoic band, whereas aged skin and photodamaged skin often show a subepidermal low echogenic band (SLEB). The presence and thickening of the SLEB are regarded as characteristic features of photoaging, and SLEB thickness is linearly associated with age. Accordingly, SLEB serves not only as a biomarker of skin aging but also as an objective endpoint for anti-aging efficacy assessment [55]. In addition, RCM can detect changes in collagen fiber network density within the superficial dermis.

The dermal matrix also contains hyaluronic acid, chondroitin sulfate, fibronectin, and other components involved in hydration, maintenance of tissue architecture, and cellular metabolism. With age, matrix hyaluronic acid content declines, and its capacity to retain water decreases, leading to reduced skin hydration and contributing to dryness and roughness [56].

Fibroblast aging is a central mechanism underlying ECM imbalance. With increasing age, the number of fibroblast decreases, and their capacity to synthesize collagen and elastic fibers is impaired. At the same time, secretion of matrix metalloproteinases, including MMP-1 and MMP-2, increases and accelerates ECM degradation. This imbalance in matrix turnover is a major driver of dermal structural aging [57].

Overall, imaging modalities such as OCT and RCM allow noninvasive discrimination of age-related histologic features in the epidermis, dermis, and skin appendages, and they have been applied in multiple anti-aging clinical trials to evaluate the efficacy of topical interventions [40]. Importantly, these techniques are limited by restricted imaging depth and field of view. In addition, collagen content and organization vary substantially among facial subregions. As a result, current imaging-based collagen quantification primarily reflects collagen status and relative change within the examined site, and its ability to represent whole-face collagen status requires further validation.

#### Subcutaneous tissue

Aging is associated with substantial changes in facial subcutaneous tissue and its supporting framework, which together drive contour alteration and tissue descent. Subcutaneous thickness progressively decreases with age, most prominently in the cheeks, temporal region, and periorbital area. Facial subcutaneous fat undergoes marked, compartment-specific remodeling with age, including selective atrophy, redistribution, descent, and focal accumulation. Loss of volume in selected midfacial compartments contributes to facial hollowing and contour changes [58, 59]. This reduction is not merely quantitative but is also accompanied by functional and morphological changes in adipocytes. Aging reduces adipocyte size and lipid storage capacity, thereby weakening structural and volumetric support. At the same time, reduced adipocyte proliferative capacity and increased secretion of pro-inflammatory mediators further aggravate degenerative changes in skin structure and function [60, 61].

In addition, deep facial retaining ligaments, including the zygomatic and mandibular ligaments, gradually become lax with age, and their connections with the skin, fat compartments, and deep fascia weaken. The combined effects of ligamentous laxity, subcutaneous fat thinning, and adipose dysfunction promote global soft-tissue descent, ultimately leading to characteristic facial aging features such as deepening of the nasolabial folds, jawline sagging, and hollowing with ptosis of the cheeks [62, 63].

#### Blood vessels and lymphatic vessels

Aging is accompanied by significant degenerative changes in both the dermal vascular and lymphatic systems, affecting nutrient delivery, immune homeostasis, and barrier maintenance.

Within the vascular system, both vessel number and function are altered with age. Vascular density decreases, especially because of capillary loss, resulting in reduced cutaneous blood supply, insufficient oxygen and nutrient delivery, and impaired cellular metabolism and repair. In parallel, vessel walls thicken, elasticity declines, fragility increases, endothelial function is impaired, and vascular permeability rises, predisposing the skin to ecchymosis, purpura, and related manifestations [64]. Cutaneous vascular changes can be assessed using several imaging approaches. High-resolution imaging and digital image analysis, including RCM, VISIA, and erythema index (EI), can provide indirect information on superficial capillary distribution. In one study conducted in a Chinese population, age-group differences in facial EI, including in the forehead and cheek regions, were not pronounced, although a slight overall decline was observed [26]. Taken together, EI appears to have relatively low specificity as an independent marker of skin aging.

Videomicroscopy and dermoscopy can also directly visualize superficial vascular morphology; for example, focal networks of dilated capillaries may be observed in photodamaged aged skin. In addition, optical coherence tomography angiography (OCT-A) and laser Doppler flowmetry (LDF) permit three-dimensional reconstruction of the cutaneous microvascular network and quantification of blood-flow density, vessel length, and branch-point number. In aged skin, microcirculation in the dermal papillae declines, accompanied by reduced vascular density and lower blood flow compared with younger skin. However, these techniques are still used mainly in research settings and are rarely part of routine clinical practice [65]. A recent study published in *Nature* further showed that regional loss of capillary-associated macrophages is a major contributor to capillary functional decline during skin aging [66].

Age-related decreases in lymphatic vessel density have also been demonstrated, particularly in both the initial and collecting lymphatic networks. This decline is closely associated with impaired lymphatic function, including reduced lymphatic pumping, diminished lymphatic return, and increased lymphatic permeability [67, 68]. At present, mature imaging and histologic tools for evaluating the cutaneous lymphatic system remain limited, and this area is still largely exploratory.

#### Skin appendages

With age, hair follicle density gradually decreases, and the hair cycle shifts toward an increased proportion of telogen follicles and a reduced proportion of anagen follicles. Hair follicles also undergo miniaturization, the bulge region that harbors hair follicle stem cells becomes smaller, and dermal papilla volume declines. At the same time, melanocyte number decreases, and melanosome synthesis and secretion are reduced, leading to thinner, lighter hair, and, eventually, hair graying [5, 69]. A cross-sectional study comparing individuals aged 20–50 years further confirmed significant age-related declines in both hair follicle density and scalp thickness, accompanied by yellowing of the hair shaft and a rougher texture [70].

Sebaceous glands may show a degree of compensatory hyperplasia in early aging, particularly in photo-exposed skin. However, with progressive aging, the glands gradually atrophy, acinar architecture becomes disorganized, and sebaceous secretion declines, contributing to skin dryness and impaired barrier function. Although existing studies have not identified consistent major age-related changes in sweat gland density or volume, three-dimensional reconstruction studies have shown that eccrine sweat gland secretory portions shift toward the skin surface with age, accompanied by increased tangling and rotation of the secretory ducts [71].

In older adults, nail aging is fundamentally driven by progressive decline in nail matrix function, manifested by reduced regenerative capacity and diminished keratinization efficiency. These changes constitute the histological basis for the macroscopic nail plate alterations described above.

It should be emphasized that the density, structure, and function of skin appendages vary substantially across anatomical sites. Moreover, most available studies are based on local regions, such as the scalp or limbs. Together with interindividual variability and the limited field of view of histologic and imaging techniques, these constraints mean that current findings primarily reflect site-specific age-related changes in appendages. Whether they can be directly extrapolated to whole-face or whole-body aging remains uncertain.

### Recommendations

1. Changes in skin tone and radiance are among the earliest visible features of skin aging, including dullness, roughness, and pigmentary abnormalities, and may serve as supplementary indicators in global aging impression scoring (Level B, Class IIa).
2. Increased wrinkle number and wrinkle depth are the most direct structural manifestations of skin aging and are widely used in population stratification and evaluation of intervention efficacy; they are priority core indicators (Level A, Class I).
3. Increased skin laxity is closely associated with reduced dermal elasticity and subcutaneous fat atrophy. Modern imaging and morphometric tools can quantify laxity effectively and are suitable for evaluating both facial and body aging (Level A, Class I).
4. Age-related changes in hair morphology, including graying, thinning, and reduced density, commonly occur with advancing age. However, because these parameters are influenced by genetics, hormones, and other factors, they are better regarded as supplementary indicators of phenotypic aging (Level A, Class IIa).
5. Nail changes are common in older adults, including thickening, longitudinal ridging, and reduced gloss. However, because standardized clinical quantification remains limited, these features are suitable only as a reference in a comprehensive assessment of phenotypic aging signs (Level B, Class IIb).
6. Structural alterations in the epidermis and DEJ are histologic correlates of age-related skin roughness and pigmentary change. Their assessment requires high-resolution imaging or biopsy-based evaluation (Level B, Class IIb).
7. Dermal thinning and collagen loss are fundamental drivers of multiple structural changes in aging skin, including laxity and wrinkles. However, direct assessment depends on staining or imaging, is constrained by sampling and imaging range, and mainly reflects local collagen status. These parameters should therefore not be used as single surrogate measures of overall skin aging, but are more suitable for mechanistic research and evaluation of intervention effects (Level B, Class IIb).
8. Thinning of the subcutaneous fat layer and laxity of supporting ligaments are manifestations of deep structural aging and may be useful for the evaluation of facial aging and, in selected settings, aging at other body sites (Level A, Class I).
9. Degenerative changes in blood vessels and lymphatic vessels are also common in aged skin, but these changes are difficult to quantify and are strongly influenced by external factors; they are therefore best considered auxiliary or research-oriented markers (Level B, Class IIb).

## Functional biomarkers

Skin aging is also accompanied by a range of physiological alterations, primarily involving decline in barrier integrity, metabolic homeostasis, elastic support, tissue repair, and immune responsiveness. These functional changes not only markedly affect the physiological state of aging skin and promote the development of age-related dermatoses, but may also increase the risk of cutaneous neoplasia, thereby substantially impairing quality of life [72, 73].

### Skin barrier function

Aging is associated with significant impairment of skin barrier integrity and water-retention capacity. These changes can be quantified using noninvasive multifunctional skin measurement devices, including transepidermal water loss (TEWL) meters, electrical impedance spectroscopy systems, corneometers, sebumeters, and pH meters [74, 75]. Common findings include reduced stratum corneum hydration and sebum secretion, together with increased TEWL and a slight increase in skin pH.

TEWL refers to the passive diffusion and evaporation of water through the stratum corneum into the external environment. It directly reflects the rate of water loss from the skin surface and is one of the most direct indicators of barrier integrity. Several studies evaluating dynamic recovery after experimental barrier perturbation indicate that baseline TEWL is not consistently elevated in aged skin. Nevertheless, following standardized challenges such as occlusion, acetone exposure, or tape stripping, aged skin shows a higher TEWL and delayed barrier recovery. These observations suggest that aging is associated with compromised barrier integrity and diminished capacity for barrier repair [75, 76]. Chronic ultraviolet (UV) exposure, as a key extrinsic factor contributing to skin aging, may further affect skin barrier function. Consistent with this notion, higher levels of sun exposure have been reported to be associated with delayed barrier recovery and increased TEWL [77]. Collectively, these findings suggest that skin aging does not necessarily manifest as a generalized elevation in baseline TEWL, but rather as a decline in barrier resilience and repair/recovery capacity, which may become more evident under occlusive conditions, irritant exposure, chronic UV exposure, or disease states. Current evidence suggests that when measurement site, method, and environmental conditions are rigorously controlled, baseline TEWL shows limited variation across racial/ethnic and geographic populations [78]. This relative cross-population comparability supports TEWL as a comparatively robust indicator of skin barrier function.

Standardized TEWL assessment is typically performed using closed-chamber probes, such as VapoMeter, or open-chamber evaporimeters, such as Tewameter, under controlled conditions of 20°C–22°C and 40%–60% relative humidity. Sebum-rich areas, such as the forehead, should generally be avoided to minimize confounding. Owing to its noninvasive, quantitative, and sensitive nature, TEWL is widely used in anti-aging product evaluation and longitudinal monitoring of barrier function [79, 80].

Stratum corneum hydration is also markedly reduced in older adults, often by 20%–40% [81], and is accompanied by reduced skin moisturization, dryness, and fissuring [82]. Large epidemiologic studies have shown that the prevalence of dry skin in individuals older than 65 years is approximately 55%–60% [83]. Clinical methods for assessing stratum corneum hydration include capacitance-based devices (such as Corneometer; normal values are generally > 30 arbitrary units (AU), whereas values < 30 AU suggest impaired barrier status), infrared spectroscopy, and confocal Raman spectroscopy, which directly quantifies water content [74, 84]. Importantly, some studies have reported significantly higher dry-skin indices in older than younger individuals among African and Caucasian populations, whereas such differences were not evident in Chinese and Mexican populations [85]. These findings suggest that ethnicity, climate, and environmental conditions may substantially influence skin hydration. Accordingly, stratum corneum hydration is best interpreted as a complementary rather than standalone biomarker of skin aging.

Electrical impedance spectroscopy (EIS) provides an additional, noninvasive electrical dimension of barrier assessment. By sweeping alternating currents across multiple frequencies, EIS generates impedance magnitude and phase spectra that reflect stratum corneum hydration and related barrier properties. Some studies suggest that EIS may be less susceptible than TEWL to short-term disturbance by routine daily activity. Moreover, the correlation between EIS and TEWL is inconsistent across studies and settings, indicating that these techniques assess distinct aspects of barrier physiology: TEWL primarily reflects transepidermal evaporative flux, whereas EIS more strongly reflects stratum corneum hydration and tissue dielectric characteristics [86, 87].

Taken together, TEWL and hydration-related measurements represent the two principal noninvasive approaches for evaluating the water-related dimension of skin barrier aging. TEWL primarily reflects evaporative water flux and therefore more directly indicates barrier integrity and water loss. By contrast, hydration-related indices focus on stratum corneum water content and water-retention capacity and include both capacitance-based measurements and EIS-derived multifrequency electrical signatures. Because individual indicators are susceptible to variation by anatomical site, environment, and population characteristics, combined use of TEWL and hydration-related measures is preferable in practice. Such combined assessment permits cross-validation of barrier-aging phenotypes across the complementary dimensions of evaporative flux and stratum corneum hydration, and may further support the development of composite measures, such as TEWL-to-hydration ratios, to improve discrimination of barrier aging and enhance cross-center and cross-population interpretability.

Sebum secretion refers to the total amount of lipids synthesized by sebaceous glands and secreted onto the skin surface, and is essential for maintaining barrier function and skin suppleness. Sebum production rises markedly during puberty, remains relatively stable until approximately 35–40 years of age, and then declines rapidly. Sebum secretion declines significantly with age and reaches its lowest levels in individuals older than 70 years [85, 88]. Sebum composition also changes with age, including a reduction in the proportion of squalene, which may further compromise barrier performance. These changes are closely related to structural remodeling of sebaceous glands. In early aging, sebaceous glands may undergo compensatory hyperplasia, particularly in photo-exposed skin, where gland size or activity may increase by as much as 70% relative to youth. However, with advancing age, the glands progressively atrophy, lipid secretion declines, and the surface lipid film thins, thereby increasing water loss and promoting dryness and scaling [89]. Standard methods for sebum assessment include photometric quantification using Sebumeter, colorimetric analysis following collection with Sebum Tape, and indirect quantification using noninvasive skin imaging systems such as the oil-analysis module of VISIA.

Healthy adult skin is mildly acidic, typically ranging from pH 4.5–6.5. This acid mantle is maintained by free fatty acids in the surface lipid film, lactic acid in sweat, and intercellular lipids within the stratum corneum. Aging skin tends to exhibit a slight increase in pH, often reaching 6–7, with the increase being most pronounced in postmenopausal women, although findings in very old adults are not entirely consistent across studies [88]. This alkaline shift may disrupt stratum corneum cohesion and accelerate barrier deterioration. Skin pH may therefore serve as a supportive biomarker of barrier aging. It is typically measured by direct skin-surface contact using a glass-electrode pH meter, such as Skin-pH-Meter. To avoid transient disturbance, measurements should not be taken immediately after cleansing; a waiting period of approximately 30 minutes is recommended.

Overall, barrier dysfunction is a major functional hallmark of skin aging and can be quantified using noninvasive instrumentation. Measurements should be performed under standardized environmental conditions, with stable probe-skin contact, to ensure reproducibility. Among currently available parameters, TEWL is the most robust core barrier indicator. By contrast, stratum corneum hydration and pH are more susceptible to environmental humidity, temperature, and individual conditions such as atopic dermatitis, and therefore have lower specificity as aging biomarkers. Nevertheless, they remain attractive because they are simple, noninvasive, and relatively inexpensive. In clinical and population-based studies, the integrated assessment of TEWL, skin hydration, sebum secretion, and skin surface pH, rather than the evaluation of these parameters in isolation, may provide a more comprehensive and accurate characterization of skin aging.

### Skin elasticity and mechanical properties

Skin elasticity is the capacity of the skin to recover rapidly after deformation induced by external force, reflecting its ability to respond to mechanical stress. In aged skin, this property is significantly impaired: even under mild external force, both the rate and extent of recoil are reduced, compromising resistance to mechanical injury. Clinically, this decline can be appreciated by delayed recoil following release of mechanical deformation. Instrument-based quantification is commonly performed using suction-based devices such as the Cutometer, which measure immediate and delayed deformation-recovery characteristics and generate parameters such as immediate elasticity (R7). Decline in these parameters is closely associated with the degree of skin aging and has become an important indicator of functional deterioration.

Studies have shown that the Cutometer-derived biological elasticity parameter R7, which reflects elastic recovery capacity, is significantly negatively correlated with age. In facial skin measurements from women, the correlation coefficient for R7 is approximately −0.62 [90]. The Cutometer is relatively simple to use, has good reproducibility, and reference values have been established in several populations. However, loss of elasticity differs by anatomical site; for example, sun-protected regions such as the inner upper arm may show slower elastic decline than the face. Ultrasound-based techniques, including acoustic radiation force impulse imaging (ARFI) and shear wave elastography (SWE), can further assess mechanical properties across the full skin thickness, from epidermis to dermis and even subcutaneous tissue. Unlike the Cutometer, which primarily reflects superficial biomechanical properties, SWE can provide depth-resolved Young’s modulus values (kPa) and display the spatial distribution of tissue stiffness using color maps. In a study of 71 women, SWE-derived dermal elasticity decreased significantly with age, and age-stratified reference values for facial skin elasticity were established [91].

### Thermoregulatory function

The skin contributes to thermoregulation primarily through vasoconstriction, vasodilation, and sweat secretion, enabling maintenance of body temperature and adaptation to environmental thermal stress.

#### Sweat gland function

In older adults, the responsiveness of sweat glands to thermal and sympathetic stimulation is reduced. Under heat stress, sweating is delayed and sweat output is significantly lower than in younger individuals [92]. Reduced sweating also weakens the moisturizing and antimicrobial functions of the skin surface, which may partly explain the dryness, reduced hydration, and increased infection susceptibility observed in aging skin. Clinically, reduced sweating may be evident in daily life, for example, diminished sweating after exercise or localized anhidrosis. Eccrine sweat gland function can also be assessed using techniques such as sweat imprint analysis and sweat-volume measurement.

#### Vascular function

Cutaneous vascular function refers to blood perfusion and vasomotor regulation within the dermal microcirculation and constitutes another major component of skin thermoregulation. In older adults, vascular elasticity declines, skin blood flow is reduced, and vascular reactivity is blunted, with delayed responses to thermal or mechanical stimuli [93]. Based on the regression relationship between age and skin blood flow, skin blood flow at 70 years of age was estimated to be approximately 40% of that at 20 years of age [94]. Methods for evaluating cutaneous microvascular function include laser Doppler flowmetry for quantifying skin perfusion and acetylcholine iontophoresis combined with laser Doppler monitoring for assessing endothelium-dependent vasodilator responses [95].

Because cutaneous microcirculation is influenced by multiple factors, including blood pressure, smoking, diet, and comorbid disease, these confounders must be considered when vascular function is evaluated as an aging biomarker. Standardized environmental conditions are recommended, and individuals with acute inflammation, diabetes, or other major confounding diseases should be excluded when possible. Combining vascular assessments with skin-response markers such as erythema index (EI) and circulating biomarkers such as senescence-associated secretory phenotype (SASP)-related factors may improve specificity and interpretability.

### Skin sensory function

#### Pain and temperature sensation

In older adults, the density of cutaneous sensory nerve endings decreases and neural function may deteriorate, often resulting in reduced sensory acuity and elevated pain and temperature thresholds. These changes increase susceptibility to low-temperature burns and traumatic ulceration [96].

#### Pruritus

Owing to dryness, impaired barrier function, and increased susceptibility of nerve endings to environmental stimuli, older adults frequently experience localized or generalized pruritus, often referred to as senile pruritus. When pruritic dermatoses coexist, patients may readily enter an itch-scratch-itch cycle [97]. Recurrent unexplained dermatitis or pruritus in older adults should therefore raise consideration of the combined effects of barrier aging and neural degeneration.

#### Skin sensitivity

Skin sensitivity refers to the tendency of the skin to develop subjective discomfort, including stinging, burning, or itching, or objective irritant responses, such as erythema, following exposure to external stimuli including cosmetics, friction, and changes in temperature or climate. Review data suggest that overall irritant sensitivity tends to decline with age [98]. This age-related decline in irritant sensitivity may reflect reduced cutaneous small-fiber innervation, elevated sensory thresholds, and impaired primary afferent function in older skin [99]. Clinical evaluation includes subjective questionnaires, the lactic acid sting test, and objective assessment such as erythema index measurement with Mexameter. However, because age-related changes in skin sensitivity do not show a clear linear relationship and are strongly influenced by interindividual variation, environmental exposure, and concomitant skin disease, skin sensitivity is not an ideal standalone marker of aging severity.

### Immune surveillance function

Cutaneous immune surveillance is the capacity of the skin immune system to maintain tissue homeostasis by recognizing, capturing, and eliminating pathogens and abnormal cells, including transformed cells. In aged skin, reduced antigen presentation and impaired immune surveillance increase susceptibility to infection, delay pathogen clearance, and elevate tumor risk [100]. For example, the risk of herpes zoster resulting from reactivation of latent varicella-zoster virus increases markedly with age, reaching approximately 6–8 cases per 1000 person-years at 60 years of age and 8–12 cases per 1000 person-years at 80 years of age [101]. The risk of premalignant lesions, such as actinic keratosis, and malignant cutaneous neoplasms, including squamous cell carcinoma, basal cell carcinoma, and melanoma, is also significantly increased in older adults [102]. Evaluation of immune surveillance typically requires invasive sampling of cells or tissue followed by immunolabeling to characterize immune cells and molecular markers. The principal noninvasive functional approach currently available is assessment of the cutaneous contact hypersensitivity response to 2,4-dinitrochlorobenzene (DNCB) [103].

### Wound-healing capacity

The capacity of the skin to repair injury declines substantially with age. Clinically, chronic ulcers, including venous leg ulcers and diabetic foot ulcers, are common in older patients and partly reflect age-related impairment in healing capacity.

Wound healing comprises three overlapping phases: inflammation, proliferation, and remodeling. Aging adversely affects each of these stages. First, the acute inflammatory phase is prolonged and functionally less efficient in older individuals. Following injury, the inflammatory response in aged skin is dysregulated, with altered neutrophil and macrophage dynamics that impair early clearance of pathogens and debris [104, 105]. At the same time, however, baseline levels of pro-inflammatory mediators are higher in aged skin, meaning that once inflammation is initiated it may persist, and sustained low-grade inflammation may damage newly formed tissue [106, 107]. Second, during the proliferative phase, fibroblast and keratinocyte proliferation and migration are reduced, resulting in delayed formation of granulation tissue and neoepidermis [105]. In addition, perfusion at wound sites is relatively poor in older individuals, and the local microenvironment is often hypoxic [108]. Finally, during remodeling, collagen reorganization and epithelial maturation are prolonged, and scar quality is poorer in aged skin [109]. As a consequence, older adults are at substantially greater risk of infection and ulceration due to chronic nonhealing wounds [109].

The reduced reparative capacity of aged skin is multifactorial. In addition to intrinsic decline in cellular proliferative potential, stem-cell exhaustion and cellular senescence contribute importantly. Epidermal stem-cell numbers in the basal layer decline with age, while functionally senescent cells accumulate and secrete inflammatory mediators that interfere with normal repair [110]. In addition, systemic conditions that are more common in older adults, such as diabetes, malnutrition, and immune dysfunction, may further delay wound healing. Accordingly, when wound healing is considered as a biomarker of skin aging, other disease-related confounders should be excluded as far as possible, and attention should be focused on baseline age-associated decline in otherwise healthy older adults. In routine clinical practice, it is not feasible to create wounds in healthy individuals to test healing capacity; however, past medical history may provide indirect insight into repair function. Minor-injury models have been used in research settings, but their application remains largely limited to experimental studies.

Overall, impaired wound-healing capacity is an important functional manifestation of organismal aging and is particularly evident in the skin. It indicates that older adults generally require more active clinical management after injury and experience longer recovery times than younger individuals. Although wound-healing capacity is not suitable for routine assessment of aging severity, it remains highly relevant in mechanistic research and geriatric care.

### Recommendations

1. Impaired skin barrier function (TEWL, stratum corneum hydration, sebum secretion, and skin pH) can serve as an important indicator of skin aging severity. Due to its high accessibility and reproducibility, it is recommended for integrated assessment (Level A, Class I).
2. Decline in skin elasticity and mechanical responsiveness is one of the most representative changes during aging. Reduced elasticity not only reflects impaired dermal structural support but is also closely associated with phenotypic features such as wrinkles and laxity, and is therefore recommended as a core assessment metric (Level A, Class I).
3. Impaired thermoregulatory function, manifested by reduced sweat secretion and blunted cutaneous blood-flow responses, is common in older adults and may be used as an auxiliary physiological functional indicator (Level B, Class IIb).
4. Changes in skin sensory function, such as elevated pain thresholds, are common in older adults but are strongly influenced by comorbid conditions and have limited specificity; they are therefore more suitable as adjunctive indicators in symptom-based assessment (Level A, Class IIa).
5. Impaired cutaneous immune surveillance in older adults is associated with increased susceptibility to skin infection and may contribute to a higher risk of certain premalignant and malignant cutaneous lesions, particularly in chronically photo-exposed skin. However, because reliable clinical quantification remains limited, this parameter is more suitable for overall health-risk assessment than as an independent biomarker (Level B, Class IIb).
6. Delayed wound-healing capacity reflects age-related impairment in tissue repair, but because standardized and ethically acceptable routine assessment is lacking, it is not recommended as a routine standalone skin-aging biomarker and should be reserved for mechanistic studies or selected clinical contexts (Level C, Class III).

## Humoral biomarkers

In the assessment of skin aging, skin interstitial fluid, blood, and microbiome-related signatures are increasingly recognized as important biomarker sources because they can be sampled in a minimally invasive or noninvasive manner, are amenable to sensitive detection, and allow quantitative analysis. Based on recent advances in skin-aging biomarker research, this consensus identifies a series of candidate fluid-related biomarkers that are closely associated with the degree of skin aging and may have predictive value, with the goal of supporting clinical assessment and monitoring of intervention efficacy. In practical application, particular attention should be paid to biomarker specificity, and potential confounding by systemic disease or external stress should be carefully excluded to ensure accurate interpretation.

### Skin-derived molecular biomarkers

With advancing age, senescent cells accumulate progressively in the skin and secrete a range of pro-inflammatory cytokines and chemokines collectively referred to as the SASP [111], including IL-1α/β, IL-6, and TNF-α [112, 113]. These mediators are derived primarily from epidermal keratinocytes [85], melanocytes [114], and dermal fibroblasts [57], and contribute to chronic low-grade inflammation within the local cutaneous microenvironment. Recent single-cell atlas studies of human skin have further shown that reduced epidermal expression of Krüppel-like factor 6 (*KLF6*) is closely associated with increased inflammatory mediators such as IL-6, whereas reduced fibroblast expression of Hairy and Enhancer of Split 1 (*HES1*) amplifies inflammatory signaling. *KLF6* and *HES1* may therefore represent potential molecular indicators of SASP activation in skin cells [41].

#### Senescence-associated secretory phenotype

SASP is widely regarded as a major driver of skin aging. Increased SASP activity can accelerate dermal ECM degradation, induce MMP expression, and impair barrier architecture, ultimately aggravating epidermal barrier dysfunction and aging phenotypes [115]. Accordingly, elevated SASP mediators in local skin interstitial fluid may serve as important biomarkers of inflammatory skin aging. However, interpretation requires caution, as local SASP levels may also be transiently elevated by acute infection, active inflammatory dermatoses, or marked environmental stress. These confounders should be excluded, and systemic inflammatory background should be considered.

#### Matrix and adhesion molecule biomarkers

The morphological and molecular basis of skin aging includes both progressive impairment of epidermal barrier structural proteins and loss or degeneration of dermal collagen and elastic fibers. Together, these changes underlie visible aging manifestations such as dryness, laxity, and wrinkles.

With age, adhesive interactions among basal keratinocytes weaken, and the expression of key junctional molecules, including E-cadherin (*CDH1*), desmocollin 3 (*DSC3*), and laminin subunit alpha-5 (*LAMA5*), gradually declines, leading to loosening of epidermal architecture and impaired barrier function [116]. This process accelerates noticeably after 60 years of age and is particularly prominent in photo-exposed skin. By contrast, individuals with a younger skin appearance tend to retain more intact epidermal organization.

Age-related changes in dermal structural proteins are also major hallmarks of skin aging. The abundance of type I and type III collagen declines significantly with age, accompanied by downregulation of collagen synthesis genes such as *COL1A1* and *COL3A1* and upregulation of MMPs and related degradative pathways, resulting in enhanced collagen breakdown and secondary disruption of basement membrane structure [117–120]. Clinical studies have shown that retinoic acid can reduce collagen degradation by suppressing *MMP-2* expression and thereby improve photoaging-associated fine lines, suggesting that MMP-2 may have value as a predictive biomarker of anti-aging treatment response [121]. Elastin, encoded by *ELN*, is a key determinant of skin elasticity. In photoaged skin, elastin commonly exhibits abnormal accumulative degeneration, namely solar elastosis, together with structural fragmentation. Available studies indicate that the decline in elastic fiber homeostasis during both intrinsic aging and photoaging is largely driven by reduced *ELN* expression, which contributes directly to loss of elasticity and wrinkle formation. Fibrillin-1, another critical structural protein, plays a central role in maintaining dermal reticular organization and mechanical stability, and has been proposed as a potential biomarker for tracking skin-aging progression and anti-aging intervention efficacy [122–124].

Despite their biological relevance, these molecules remain at an early translational stage, and robust large-scale multicenter clinical validation is still lacking. Future prospective cohort studies are needed to determine their reproducibility, accuracy, and practical applicability. In addition, these markers may also be influenced by non-aging processes such as wound repair, inflammation, or scar formation, and should therefore be interpreted in conjunction with histologic and functional skin assessments.

#### Oxidative stress and antioxidant biomarkers

Skin aging is characterized by a dynamic imbalance between enhanced oxidative stress and weakened antioxidant defense. Ultraviolet irradiation and cellular metabolism promote the accumulation of reactive oxygen species (ROS) in the cutaneous microenvironment, thereby activating multiple pro-aging pathways, including suppression of collagen synthesis, activation of MMPs, and induction of SASP secretion [125].

Among the best-characterized markers of oxidative damage in skin tissue are 8-hydroxy-2'-deoxyguanosine (8-OHdG) and malondialdehyde (MDA). 8-OHdG is a classical marker of oxidative DNA damage and is significantly elevated in both photoaged and intrinsically aged skin [126, 127]. MDA, an end product of lipid peroxidation, is also increased under ultraviolet exposure and during physiologic aging, and is considered an important indicator of oxidative injury in the skin [128, 129].

Antioxidant defense parameters may also provide useful information regarding skin aging. In young skin, antioxidant enzymes such as glutathione peroxidase (GPx) and superoxide dismutase (SOD) are generally maintained at relatively high activity levels, whereas aged skin often shows reduced or depleted antioxidant enzyme activity [130]. Clinical and experimental studies have shown that oral antioxidant plant formulations may improve photoaging phenotypes by increasing SOD and GPx activity, enhancing total antioxidant capacity, and reducing MDA and tyrosinase expression [131]. It should be noted, however, that antioxidant enzyme activity may transiently increase after acute photodamage as a compensatory response. Over the long term, aging tissues more often exhibit diminished antioxidant reserve.

Accordingly, combined assessment of oxidative damage biomarkers, such as 8-OHdG and MDA, together with antioxidant parameters, such as SOD, GPx, and glutathione-related indices, may be useful in evaluating skin aging. These markers should be interpreted in the context of ultraviolet exposure, smoking, and other lifestyle factors.

#### Hormonal biomarkers

The skin functions as a neuro-endocrine-immune organ: it is both a target of multiple hormones and a site of local hormone synthesis and metabolism, particularly within epidermal keratinocytes and hair follicles. With advancing age, endocrine axis function progressively declines, and changes in the levels of certain hormones and their metabolites within skin tissue are closely associated with skin aging.

Studies have shown that cortisol concentrations in skin are significantly higher in older adults than in younger individuals [112]. Cortisol is a prototypical stress hormone, and chronic elevation can promote degenerative structural changes including collagen loss and skin thinning [132]. Reduced sensitivity of hypothalamic-pituitary-adrenal (HPA) axis regulation with age may favor local cortisol accumulation in the skin [85]. Skin cortisol has therefore been proposed as a potential biomarker of chronic stress-related skin aging. However, because local cortisol is strongly influenced by psychological stress, sleep disturbance, and systemic medication use, it should be interpreted together with other biomarkers.

The local sex-hormone milieu is also critically involved in skin aging [133]. Dermal fibroblasts express estrogen receptors and respond to estrogenic signaling by regulating collagen synthesis and sebum metabolism [134]. In postmenopausal women, marked reduction in 17β-estradiol is accompanied by dryness, collagen loss, reduced elasticity, and deepening of wrinkles [135, 136]. Clinical studies further suggest that topical estrogen can promote collagen synthesis and improve skin thickness and hydration [137]. These findings support a role for estrogen-related parameters as candidate indicators of hormonally driven skin aging, while also underscoring the importance of sex and menopausal status in interpretation.

#### Other biomarkers

Hyaluronic acid (HA) is a major glycosaminoglycan involved in maintaining dermal structural integrity and tissue hydration. Studies have shown that cutaneous HA content declines with age, contributing to dryness, reduced elasticity, increased wrinkling, and diminished hydration [138]. As a key ECM component, HA is closely linked to skin structure and function, and its age-related decline may therefore serve as an indicator of matrix aging and impaired water-retention capacity. Although HA supplementation has been widely used in anti-aging skincare and regenerative strategies [56, 139], its value as a biomarker currently remains more supportive than definitive.

Homocitrulline (Hcit) has recently been proposed as an emerging fluid-related biomarker of skin aging. Hcit is an irreversible end product of protein carbamylation derived from lysine residues and reflects the cumulative effects of long-term oxidative stress and chronic inflammation. Studies have shown that Hcit levels are significantly elevated in both the skin and serum of older adults and in patients with seborrheic keratosis, and that Hcit correlates positively with age [140]. These findings suggest that Hcit may indicate irreversible structural degeneration of the skin.

In recent years, nucleic acid molecules derived from skin tissue, particularly small RNAs, have also been proposed as potential biomarkers of skin aging. Multiple aging-related miRNAs have been implicated in the regulation of cellular senescence, inflammation, pigmentation, and extracellular matrix homeostasis in the skin [141]. Emerging preclinical evidence further indicates that specific tsRNAs, including 5′-tiRNA-His-GTG, are dysregulated in photoaged fibroblasts and skin tissue, although their presence and biomarker value in human skin interstitial fluid remain to be established [142]. Similarly, miR-31 is markedly upregulated in both epidermal and hair follicle stem cells during aging, and genetic and pharmacologic data suggest that inhibition of miR-31 may alleviate stem-cell exhaustion and multiple skin-aging phenotypes [143]. These findings support the potential of RNA-based biomarkers, but at present they remain largely confined to basic and preclinical research and depend on laboratory platforms such as high-throughput sequencing and real-time quantitative polymerase chain reaction (qPCR). Standardized methods and large-scale population validation are still lacking.

### Blood biomarkers

#### SASP markers

SASP exerts pro-inflammatory effects not only locally but also systemically, and elevated circulating SASP-related factors are an important feature of organismal aging. Recent studies have shown that serum levels of inflammatory mediators such as IL-6, TNF-α, and C-reactive protein (CRP) are generally elevated in older adults, and persistent elevation of these mediators is associated with impaired barrier function, altered dermal remodeling, and inflammatory aging phenotypes of the skin [144].

However, circulating SASP-related factors are not skin-specific. Their levels may be influenced by infection, chronic systemic disease, or autoimmune status, among other confounders. Therefore, when such markers are used in the context of skin aging, they should be interpreted together with skin-derived molecular markers, imaging findings, functional parameters, and the broader clinical background in order to improve specificity and reduce misclassification.

#### Oxidative stress and antioxidant biomarkers

Frequently used circulating oxidative stress markers include MDA, 8-OHdG, and SOD, and changes in these markers are closely associated with skin aging [145]. Because the skin is directly exposed to oxidant stressors such as ultraviolet radiation, systemic decline in antioxidant capacity may increase susceptibility to photoaging and pigmentation disorders. Clinical and animal studies have shown that antioxidant plant-derived supplements can reduce serum MDA and improve antioxidant status, thereby ameliorating skin elasticity and pigmentation [131]. In addition, one study found that intrinsic skin autofluorescence intensity in photo-exposed skin was negatively correlated with serum MDA levels, suggesting that skin autofluorescence may represent a noninvasive surrogate measure of systemic oxidative injury relevant to skin aging [146].

Thus, circulating oxidative stress and antioxidant markers may provide a systemic perspective on skin aging by reflecting not only cutaneous oxidative injury but also whole-body redox balance. In practice, such systemic markers are best used as adjuncts to local skin-derived measures rather than as standalone indicators.

#### Hormonal and metabolic biomarkers

Multiple studies have demonstrated that hormonal fluctuations play a key regulatory role in skin aging. Estrogen promotes skin thickness and hydration, and estrogen deficiency is considered an important contributor to dryness and atrophy in perimenopausal women [133, 147]. Reduced testosterone in older men is also associated with lower sebum secretion and impaired wound-healing capacity [148]. Melatonin, a pineal circadian hormone, has antioxidant, anti-inflammatory, and DNA repair-promoting properties. Its serum levels decline with age and have been shown to correlate negatively with skin-aging phenotypes such as wrinkle scores [149]. Nevertheless, despite their biological relevance, these hormones have important limitations as skin-aging biomarkers because they are influenced by systemic physiology, and circadian rhythm and have limited organ specificity.

The role of growth hormone (GH) and insulin-like growth factor 1 (IGF-1) in skin aging is more complex. GH and IGF-1 may be produced locally by dermal fibroblasts and other cutaneous cells, and contribute to stem-cell maintenance, collagen synthesis, and barrier homeostasis [150–153]. A notable example is Itabaianinha syndrome, caused by growth hormone-releasing hormone receptor mutations leading to persistently low GH/IGF-1, in which the skin exhibits marked wrinkling, underscoring the importance of GH/IGF-1 signaling in skin-aging regulation [154]. Studies have shown that individuals with relatively higher serum IGF-1 tend to have fewer wrinkles [155], but excessively high IGF-1 may also increase the risk of certain age-related diseases, including malignancy. Thus, IGF-1 should be interpreted bidirectionally in skin-aging assessment: low levels may reflect insufficient anabolic and regenerative signaling, whereas excessively high levels may indicate abnormal proliferative risk.

Dehydroepiandrosterone (DHEA) is an endogenous steroid hormone that peaks between 20 and 30 years of age and then declines progressively. Reduced DHEA has been associated with skin dryness and loss of elasticity, supporting its potential role as an age-related endocrine marker of skin aging [156].

Metabolic status also contributes importantly to skin aging. Population studies have shown that hyperglycemia is associated with an aged appearance, including dull skin tone and deeper wrinkles, which may be related to accumulation of advanced glycation end-products (AGEs) in the dermis and consequent disruption of collagen architecture [157]. In addition, Mendelian randomization analysis has suggested a significant causal relationship between blood fatty acid unsaturation and skin-aging risk, with higher levels of unsaturation, particularly docosahexaenoic acid (DHA) and polyunsaturated fatty acids (PUFAs), potentially accelerating skin aging [158]. These observations suggest that circulating metabolic profiles may provide additional insight into skin-aging risk.

Overall, circulating hormone levels and metabolic profiles offer a systemic perspective on skin aging. Their value lies not only in reflecting skin status but also in informing broader endocrine or metabolic intervention strategies. Interpretation should always take into account age, sex, physiologic rhythm, and the possibility of confounding by systemic disease.

### Microbial-related biomarkers

The skin and gut microbiota regulate organismal aging through multiple pathways and affect barrier function, immune responses, oxidative stress, cellular metabolism, and visible skin phenotypes. Increasing evidence indicates that the composition and functional capacity of microbial communities change significantly with age. These shifts may not only reflect age-related changes in skin physiology but may also contribute to the pathogenesis of skin aging, thereby supporting their potential role as biomarkers.

#### Skin microbiota

The microbial community on the skin surface evolves dynamically across the lifespan. Dominant commensals such as *Cutibacterium acnes* predominate in young and middle-aged skin, but their relative abundance declines significantly in older skin. By contrast, *Corynebacterium*, *Staphylococcus*, and *Micrococcus* tend to increase, with an overall pattern of greater microbial diversity but reduced ecological stability [159, 160]. These compositional shifts are closely associated with physiologic changes including reduced sebum secretion, impaired barrier function, and increased inflammatory susceptibility [161, 162].

At the functional level, the metabolic capacity of the skin microbiome also changes with age. Younger skin is enriched in microbial pathways related to vitamin synthesis, cofactor metabolism, and antioxidative functions, whereas older skin tends to show enrichment of lipid metabolism and stress-response pathways [163]. Loss of microecological stability may disrupt microbial interaction networks, permit expansion of opportunistic strains, and reduce antioxidant metabolite production by beneficial microbes, thereby accelerating skin aging, particularly under extrinsic stressors such as ultraviolet exposure and environmental pollution [164]. Psychological stress may further activate the HPA axis, increase cortisol secretion, alter skin microbial composition, and exacerbate local inflammation, forming a “stress-skin-microbiome axis” that may further promote skin aging [165].

Notably, specific skin microbial features, including taxonomic and functional α-diversity, as well as the relative abundance of key species such as *Moraxella osloensis*, *Streptococcus gordonii*, *Cutibacterium acnes*, and *Corynebacterium matruchotii*, have been associated with visible imaging traits such as pigmentation, wrinkles, enlarged pores, sebum, and skin gloss. These microbial signatures have been incorporated into “microbial clock” models to estimate biological age and aging severity [166].

In summary, skin microbial features, such as community diversity and dominant taxa abundance, may have value in monitoring skin aging and estimating biological age. However, this field remains exploratory, and more longitudinal studies and large-scale population datasets are required before clinical implementation can be considered.

#### Gut microbiota

The gut microbiota is another major microecological network relevant to skin health. With age, beneficial organisms such as *Bifidobacterium* and butyrate-producing bacteria decline, whereas the proportion of potentially pathogenic bacteria increases, leading to impaired intestinal barrier function and persistent chronic low-grade inflammation. Through the gut-skin axis, this systemic inflammatory state may impair cutaneous immunity, metabolism, and regenerative capacity, thereby accelerating skin-aging phenotypes [167, 168].

Studies have shown that modulation of gut microbial ecology, for example through fecal microbiota transplantation or functional dietary intervention, can improve skin barrier structure, enhance collagen synthesis and anti-inflammatory factor expression, and thereby delay skin aging [168, 169]. Certain gut-derived metabolites, such as L-arginine and L-ornithine, may exert systemic effects through mitogen-activated protein kinase (MAPK) signaling and regulation of skin-aging-related genes including aquaporin 3 (*AQP3*) and filaggrin (*FLG*) [168]. However, the causal roles and functional relevance of specific gut microorganisms and metabolites in skin aging remain insufficiently defined. Their utility as functional biomarkers therefore requires further mechanistic and longitudinal validation.

### Recommendations

1. Expression of SASP factors in skin tissue, such as IL-1α/β, IL-6, and TNF-α, is associated with skin aging but has not yet entered clinical use. These markers are recommended as candidate fluid-related biomarkers for skin-aging assessment, provided that the influence of other skin diseases is excluded (Level B, Class IIa).
2. Reduced expression of epidermal adhesion proteins in skin tissue, including CDH1, DSC3, and LAMA5, suggests age-related deterioration of epidermal structure and may serve as a potential marker of declining barrier function, provided that other causes of barrier damage are excluded (Level B, Class IIa).
3. Reduction of dermal structural proteins, including type I and III collagen, is associated with skin aging but has not yet been clinically implemented, and may be considered a candidate biomarker related to skin aging (Level B, Class IIa).
4. Increased levels of oxidative stress markers, such as 8-OHdG and MDA, in skin tissue and serum are associated with skin aging and may serve as systemic biochemical indicators for skin-aging assessment (Level B, Class IIb).
5. Hcit levels in skin tissue and serum may serve as candidate biomarkers of skin aging; elevated levels reflect chronic oxidative stress and irreversible tissue degeneration and may indicate the presence of skin aging (Level B, Class IIb).
6. Reduced serum estrogen suggests decreased skin thickness and impaired barrier function and may therefore indicate skin aging; however, because this marker is not skin-specific, overall organismal aging should be considered in interpretation (Level B, Class IIb).
7. Serum melatonin may serve as a metabolic biomarker for skin aging; lower serum melatonin levels are associated with greater wrinkle formation and may indicate skin aging (Level B, Class IIb).
8. Serum IGF-1 may serve as an auxiliary biomarker for assessing the degree of skin aging, particularly in relation to facial wrinkles and tissue structural changes (Level B, Class IIb).
9. Blood glucose should not be used as a standalone metabolic biomarker for skin-aging assessment, given its limited specificity and clinical value (Level B, Class III).
10. Circulating unsaturated fatty acids, such as DHA and PUFAs, are not recommended as routine metabolic biomarkers of skin aging at present; although higher levels have been associated with increased skin-aging risk, their specificity and clinical applicability remain insufficient (Level B, Class III).
11. Skin microbiota characteristics, including *Cutibacterium* abundance and microbial diversity, are not recommended as routine biomarkers for individual skin-aging risk assessment at present; despite their exploratory value for estimating biological age and aging severity, current evidence remains insufficient for clinical implementation (Level B, Class III).

## Construction of “skin age” prediction models

As the largest organ of the human body, the skin undergoes aging through comprehensive, multiscale alterations spanning molecular changes, such as collagen degradation, telomere shortening, and epigenetic modification; cellular changes, including fibroblast and keratinocyte senescence and stem-cell exhaustion; tissue-level remodeling, including epidermal and dermal thinning, subcutaneous fat atrophy, and disorganization of collagen and elastic fibers; functional decline, such as impaired barrier integrity, delayed wound healing, and altered immune responsiveness; and visible phenotypic changes, including wrinkles, pigmentary lesions, laxity, and dryness. These changes not only affect appearance but also make the skin an important window into organismal aging, and they are associated with increased risk of multiple skin disorders, including actinic keratosis and cutaneous malignancy. Accurate assessment of skin biological age, hereafter referred to as SkinAge, together with prediction of its trajectory and associated disease risk, requires integration of multidimensional and multiscale information. In recent years, advances in artificial intelligence and multi-omics technologies have enabled initial progress in SkinAge prediction research.

### Standardization of measurement methods and acquisition workflows

Before SkinAge models can be robustly developed, measurement methods and acquisition workflows should be further standardized to establish a cross-center data collection framework that is comparable, reproducible, and reusable.

Noninvasive assessments are well suited to large cohorts and longitudinal follow-up and generally offer good repeatability, but they are susceptible to environmental conditions, operator variation, and device differences, and they have limited capacity to resolve deeper structures. These approaches mainly include facial and skin imaging or optical imaging, such as multisource digital photography, three-dimensional surface profilometry, dermoscopy, RCM, OCT, and high-frequency ultrasound, as well as physiologic functional measurements, including TEWL, stratum corneum hydration, EIS, sebum, pH, suction-based elasticity testing, and ultrasound elastography. By contrast, minimally invasive or invasive methods can provide higher information density and mechanistic insight, but they impose greater demands on quality control and may introduce sampling-related bias. These approaches mainly include blood sampling and skin-sample or microbiome-related analyses.

From the perspective of signal origin, currently available methods can be broadly categorized into five physical dimensions: mechanical, thermal, acoustic, optical, and electrical. Mechanical measurements primarily reflect surface topography and biomechanical response, including wrinkles, laxity, and elasticity parameters, and are therefore closely linked to visible appearance and tactile changes. Thermal measurements focus on body temperature and sweat-related phenotypes, but are highly sensitive to ambient conditions and recent physical activity. Acoustic approaches, represented by high-frequency ultrasound, vascular ultrasound, and ultrasound elastography, provide structural and blood-flow information from deeper tissue layers, but are subject to operator dependence. Optical approaches, including spectrocolorimetry, multisource imaging, and RCM/OCT, offer high spatial resolution and quantitative assessment of structural and appendage-related features, although they remain limited by field of view and imaging depth. Electrical approaches, such as EIS and pH measurement, primarily capture barrier-related electrophysiologic phenotypes and may serve as complementary readouts to water-related metrics such as TEWL and stratum corneum hydration, thereby improving the robustness of barrier assessment.

To achieve quantitative and phenotypic discrimination between intrinsic aging and ultraviolet-driven extrinsic aging, it is necessary to establish a normative baseline database of skin aging in the Chinese population covering the full age range from 1 to 100 years. Cohort design should include both exposed and non-exposed sites, ideally with within-subject paired comparisons, and should systematically record key extrinsic exposures, including sun exposure and photoprotection behaviors, occupation and outdoor activity, air pollution, and smoking. This framework would facilitate separation of intrinsic aging trajectories from superimposed extrinsic effects, particularly those driven by light exposure, and would provide a reference for phenotypic classification and intervention assessment.

Under conditions that balance scalability and cost control, a unified multicenter core noninvasive acquisition workflow should be prioritized. First, noninvasive testing should be implemented as the multicenter minimum essential dataset, including barrier and hydration indices, such as TEWL, stratum corneum hydration with or without EIS, sebum, and pH; biomechanical measures, such as elasticity and rebound parameters; and appearance-related pigmentary and vascular phenotypes, including erythema, melanin or pigmented area, wrinkle depth or number, and laxity, assessed using multisource imaging and/or three-dimensional surface profilometry. High-frequency ultrasound may be added where appropriate to assess subcutaneous fat thickness, and laser Doppler methods may be incorporated for microcirculation assessment.

Second, molecular testing should include blood-based and microbiome-related indicators to provide information on systemic aging and metabolic or inflammatory background. Blood collection windows should be standardized, preferably in the fasting morning state or at a fixed postprandial interval, together with anticoagulant tube type, centrifugation protocol, cryopreservation workflow, and microbial sampling conditions.

Third, cellular and tissue assays may be incorporated as an enhanced module under appropriate ethical oversight for feature interpretation and biomarker validation. These may include dermal collagen and elastic fiber indices, such as collagen density or content, type III collagen, and elastin; cell-level phenotypes, such as fibroblast number and activity-related readouts; and SASP-related factors. Sampling site, sampling depth, and timing should be strictly standardized and paired with noninvasive measurements on the same day or within a prespecified acquisition window.

Fourth, internationally recognized standardized questionnaires should be used in parallel to collect key covariates, including sun exposure and photoprotection, smoking and alcohol consumption, occupation and outdoor exposure, sleep and diet, medication history, comorbid disease, and previous dermatologic treatment or surgery, for stratification and model adjustment. All measurements should be conducted under unified standard operating procedures, including fixed acquisition times, standardized ambient temperature and humidity, controlled rest periods, defined requirements for skin cleansing and discontinuation of topical products, and standardized illumination, camera distance, head position, and facial expression during facial image acquisition.

### Skin aging assessment models and prediction models for skin aging-related diseases

SkinAge is intended to quantify the true degree of aging in an individual’s skin structure and function, thereby overcoming the limitations of chronological age and more precisely addressing the question of how old the skin biologically is at a given time. The skin-aging biomarkers described above, spanning molecular, cellular, tissue, functional, imaging, and visible phenotypic domains, provide a broad range of quantitative features for model construction. Conversely, a robust SkinAge model may itself serve as a practical reference standard for biomarker selection and validation.

At present, clinical assessment is based mainly on imaging modalities such as facial photography, reflectance confocal microscopy, and confocal optical coherence tomography, with noninvasive evaluation achieved by quantifying phenotypic features including wrinkles, pores, and pigmentary lesions [170]. In recent years, artificial intelligence has accelerated the use of machine-learning methods for estimating physiologic skin age. On the basis of existing functional and imaging measurements, algorithms such as artificial neural networks (ANNs) and convolutional neural networks (CNNs) have been used to develop and validate skin-aging prediction models [171]. For example, a recent CNN-based model trained on approximately 5000 noninvasive three-dimensional facial images from Han Chinese individuals achieved mean prediction differences of 2.8 years for chronological age and 2.9 years for perceived age [172]. Further analysis of blood transcriptomes from 280 individuals, combined with causal inference modeling, was used to identify molecular regulators through which lifestyle may influence the rate of facial aging [172].

More recently, the ThermoFace model, based on thermal infrared facial imaging, has been proposed as a novel approach for both skin-aging and metabolic-health assessment. This study collected thermal infrared facial images from 2811 Han Chinese participants and, following facial landmark matching and standardized preprocessing, developed three aging-prediction models: partial least squares regression (PLSR), elastic net (EN), and CNN. Among these, the CNN model performed best, with a mean absolute error of 5.08 years, and retained stable performance in an independent cohort. A key strength of ThermoFace lies in its close association with metabolic state and molecular pathways. Its adjusted age gap was positively correlated with body mass index (BMI), fasting glucose, and low-density lipoprotein, and was linked to blood transcriptomic pathways related to DNA repair, lipolysis regulation, and ATPase activity. At the same time, the model showed strong predictive performance for metabolic disorders, including fatty liver in men, with an area under the curve (AUC) of 0.892, and hypertension in women, with an AUC of 0.862, and was also able to dynamically reflect the effects of exercise intervention. However, ThermoFace still has important limitations: the current dataset includes only Han Chinese participants, largely from climatically similar regions such as Hebei and Beijing, and lacks validation across ancestry groups and diverse climate zones. Long-term follow-up validation is also still needed [173].

Recent advances in deep learning have further expanded interest in facial image-based biological age estimation. The FaceAge model, trained on facial image data from 58,851 individuals aged 60 years and older who were considered healthy, can estimate biological age from facial photographs and has shown potential utility in survival prediction among patients with cancer [174]. However, this model also has substantial limitations. Its training data are composed primarily of White faces, and the extent to which cosmetic procedures, makeup, lighting, and facial angle influence model output remains unclear. In addition, large-scale cohort validation and long-term follow-up are still lacking.

High-throughput omics technologies, including whole-genome profiling, transcriptomics, noncoding RNA analysis, DNA methylation analysis, metabolomics, and proteomics, have also been widely applied to aging research. Because skin is one of the few organs from which tissue can be obtained relatively easily, it provides a practical substrate for biomarker discovery and age-prediction modeling. A recent study used DNA methylome data from 508 human skin samples to construct a skin-specific age predictor based on 2266 cytosine-phosphate-guanine (CpG) sites. This model effectively predicted both skin health status and DNA methylation age and showed improved precision and lower standard deviation than previous DNA methylation age predictors [175]. In addition, emerging technologies such as single-cell or single-nucleus sequencing, spatial transcriptomics, and spatial metabolomics may enable discovery of more specific indicators of skin biological age. However, the large volume and complexity of these data make SkinAge assessment costly and difficult to scale.

Compared with aging models for other organs, skin-aging prediction models face distinctive challenges. Environmental exposures, particularly ultraviolet radiation, are major extrinsic drivers of skin aging, and their effects are highly cumulative and strongly modified by individual behavior and context, including geographic location, occupation, and photoprotection habits. As a result, skin aging is more environmentally contingent and requires more complex quantitative modeling than many other organ systems. In addition, skin aging is closely linked to several skin-specific disorders, including cutaneous cancer and pre­cancerous lesions, as well as photodamage-related conditions such as seborrheic keratosis. Because aging and disease are deeply coupled at both mechanistic and phenotypic levels, distinguishing pure physiologic aging from early pathologic change is particularly challenging. Skin-aging prediction models must therefore account not only for interactions between environmental and genetic factors, but also for the complex relationship between aging and disease, in order to achieve more precise and personalized assessment.

## Conclusion and future perspectives

### Key recommendations for skin aging biomarkers

Based on expert discussions, skin aging biomarkers are recommended from three dimensions: structural biomarkers, functional biomarkers, and humoral biomarkers. These markers may have clinical applicability, but further validation in cohorts of different age groups is still needed (Table 2).

**Table 2.** Biomarker recommendations for skin aging

	Biomarker	Detection method	COR	LOE
**Structural biomarkers**	Skin tone and radiance	Skin spectrophotometric colorimeter, Erythema/Melanin meter	IIa	B
	Wrinkles	Multisource digital facial skin imaging system, 3D skin surface profilometry	I	A
	Skin laxity	Multisource digital facial skin imaging system	I	A
	Hair morphology	Dermoscopy, Automated tensile testing	IIa	A
	Fingernails and toenails	Dermoscopy	IIb	B
	Epidermal and DEJ structures	RCM, OCT, High-frequency ultrasound	IIb	B
	Collagen and elastic fibers	Multiphoton microscopy, RCM	IIb	B
	Subcutaneous fat	Ultrasound, MRI	I	A
	Blood vessels	Laser Doppler flowmetry, RCM, OCT, Dermoscopy	IIb	B
**Functional biomarkers**	Skin barrier	TEWL meter, Corneometer, EIS, Sebumeter, Skin pH meter	I	A
	Skin elasticity	Suction-based skin measurement device; Ultrasound elastography	I	A
	Skin thermoregulation	Lodine-starch test, Laser Doppler flowmeter, Vascular ultrasound	IIb	B
	Skin sensory	Tactile/Pain stimulator, Skin testing device	IIa	A
	Immune surveillance	Tissue analysis/Immunofluorescence	IIb	B
	Wound healing	Standardized small-wound healing test	III	C
**Humoral biomarkers**	SASP	ELISA (skin)	IIa	B
	Epidermal adhesion proteins	ELISA, qPCR (skin)	IIa	B
	Type I/III collagen	Hydroxyproline assay (skin)	IIa	B
	Hcit	ELISA (skin)	IIb	B
	8-OHdG, MDA	ELISA (blood), Immunofluorescence (skin)	IIb	B
	Estrogen, IGF-1	ELISA (blood)	IIb	B
	Blood glucose, Unsaturated fatty acids	Biochemical assay (blood), LC-MS/MS (blood)	III	B
	Surface microbiota	Gene sequencing (microbiota)	III	B

Note: 8-OHdG, 8-hydroxy-2′-deoxyguanosine; COR, Class of Recommendation; DEJ, dermal-epidermal junction; EIS, electrical impedance spectroscopy; ELISA, enzyme-linked immunosorbent assay; Hcit, homocitrulline; IGF-1, insulin-like growth factor 1; LC-MS/MS, liquid chromatography-tandem mass spectrometry; LOE, Level of Evidence; MDA, malondialdehyde; MRI, magnetic resonance imaging; OCT, optical coherence tomography; qPCR, quantitative polymerase chain reaction; RCM, reflectance confocal microscopy; SASP, senescence-associated secretory phenotype; TEWL, transepidermal water loss.

### Roadmap for skin aging biomarker research in China

1. To establish a national multicenter aging cohort by integrating clinical data with basic research, with the aims of discovering and validating skin aging biomarkers and constructing a reference system of skin aging biomarkers suitable for the Chinese population. Through prediction of critical inflection points in skin aging, this effort will help define individualized time windows for intervention in skin aging and related diseases, thereby providing a scientific basis for early prevention and intervention.
2. To develop skin aging prediction models based on artificial intelligence and machine learning. By integrating multidimensional information, including genetic background, living environment, and lifestyle, these models will enable prediction of skin aging at both macroscopic and microscopic levels. In combination with predictive models for skin aging-related diseases, they will support precise prediction and intervention for skin aging and its related disorders.
3. To promote synergistic development across the entire chain of industry, academia, and research, thereby accelerating the translation and application of scientific advances. Through deep collaboration among industrial, academic, and research sectors, this framework will facilitate the broad application of skin aging biomarkers and drive innovation and optimization of related technologies, ultimately improving skin health, strengthening prevention and treatment of related diseases in China, and enhancing public awareness of skin health.
